# Opioid use disorder risk alleles in self-reported assigned African American/Afro-Caribbean and European biogeographical genetic ancestry groups and in males and females

**DOI:** 10.1038/s41397-024-00337-y

**Published:** 2024-08-01

**Authors:** Jon E. Sprague, Caroline E. Freiermuth, Joshua Lambert, Robert Braun, Jennifer A. Frey, Daniel J. Bachmann, Jason J. Bischof, Lauren Beaumont, Michael S. Lyons, Michael V. Pantalon, Brittany E. Punches, Rachel Ancona, David F. Kisor

**Affiliations:** 1 Bowling Green State University, The Ohio Attorney General’s Center for the Future of Forensic Science, Bowling Green, OH USA; 2 Department of Emergency Medicine, University of Cincinnati, Cincinnati, OH USA; 3 Center for Addiction Research, University of Cincinnati College of Medicine, Cincinnati, OH USA; 4 College of Nursing, University of Cincinnati, Cincinnati, OH USA; 5 Department of Emergency Medicine, The Ohio State University, Columbus, OH USA; 6 Department of Pharmaceutical Sciences and Pharmacogenomics, College of Pharmacy, Natural and Health Sciences, Manchester University, Fort Wayne, IN USA; 7 Department of Emergency Medicine, Yale University School of Medicine, New Haven, CT USA; 8 College of Nursing, The Ohio State University, Columbus, OH USA; 9 Washington University School of Medicine, St. Louis, MO USA

**Keywords:** Biomarkers, Predictive markers

## Abstract

The influence of genetic variants related to opioid use disorder (OUD) was evaluated using multiple logistic regression analysis in self-reported assigned African American/Afro-Caribbean and European biogeographical ancestry groups (BGAGs) and by sex. From a sample size of 1301 adult patients (>18 years of age) seen in emergency departments of three medical centers in Ohio, six variants were found to be associated with OUD. Two of the variants, rs2740574 (*CYP3A4*) and rs324029 (*DRD3*), were included in the analysis having met criteria of at least five subjects for each BGAG, variant carrier status, and OUD status combinations. Variant carriers in the African/Afro-Caribbean BGAG had slightly lower predicted probabilities of OUD. Variant carriers in the European BGAG had slightly higher predicted probabilities of OUD. Relative to sex, all the six variants met evaluation criteria (five subjects for all sex, variant, and OUD status combinations). No statistically significant interactions were found between a given variant, BGAGs and sex. Findings suggest variant testing relative to OUD risk can be applied across BGAGs and sex, however, studies in larger populations are needed.

## Introduction

Opioid use disorder (OUD) is a chronic condition related to the use of opioids *“causing clinically significant distress or impairment*” and opioid overdose is an epidemic in the United States and worldwide [1, 2]. Current estimates suggest that over six million individuals in the United States have OUD [2]. In 2021, the U.S. Centers for Disease Control estimated that overdose deaths increased by approximately 15% from 2020 with more than 107,500 drug overdose deaths [3]. In sheer number, in the U.S., opioids overdose deaths in 2020 were highest among the White race/ethnicity population at just over 47,300, a 31% increase from 2019 [3]. The Black race/ethnicity population saw almost 11,000 opioid overdose deaths, representing a 55% increase from the previous year [3]. Synthetic fentanyl derivatives have been related to the highest number of overdose deaths in the U.S., with nearly 60,000 in 2021 [4].

While all-cause emergency department (ED) visits decreased in 2022, compared to 2018 and 2019, non-fatal opioid overdose visits increased nearly 11% [5]. In an effort to identify genetic risk factors of OUD, a study was undertaken in ED patients at three large urban EDs in Ohio, a state which has ranked in the top five for opioid overdose deaths since 2014 [4, 6, 7]. From June 2020 to November 2021, 1301 patients were enrolled in the study. In these patients, we examined the genetic association of 180 candidate single nucleotide polymorphisms (SNPs/variants), with 120 being pharmacodynamic-related variants and 60 being related to pharmacokinetics. Of the 1301 patients, 250 patients met the DSM-5 criteria for a diagnosis for current or lifetime OUD. Of these, 161 patients were in the European genetic ancestry group, 79 were in the African American/Afro-Caribbean genetic ancestry group, and 10 patients were not identified as being in a specific genetic ancestry group [7]. In the 250 patients who met criteria for an OUD diagnosis, a total of six variants were associated with OUD. Four single nucleotide polymorphisms (SNPs) identified in a total of two genes were related to increased risk of OUD. Homozygous SNPs in *CYP3A5*, rs15524 (A/A) and rs776746 (C/C) had the highest odds ratios at 2.812 and 2.495, respectively [7]. Additionally, two homozygous SNPs in *DRD3* (rs324029 C/C and rs2654754 T/T), with odds ratios of 2.057 and 1.462, respectively, are also related to increased risk of OUD [7]. Conversely, two SNPs were identified that were related to decreased risk of OUD. The SNPs included rs2740574 (*CYP3A4*; C/C) and rs2069514 (CYP1A2; A/A), with odds ratios of 0.471 and 0.683, respectively [7].

The six variants found to be associated with the development of OUD have both pharmacodynamic and pharmacokinetic considerations [7]. Pharmacodynamically, *DRD3* regulates the expression of the dopamine 3 receptor (D3R). The D3R is predominantly a presynaptic autoreceptor that regulates dopamine concentrations in the reward pathways [8]. Pharmacokinetically, the remaining four variants were associated with drug metabolism [7]. Homozygous SNPs in *CYP3A5*, rs15524 and rs776746 were also found to be associated with an increased risk of developing OUD. CYP3A5 has been identified to play a role in the metabolism of opioids [9] and reduced function SNPs could result in higher plasma concentrations of opioids [9]. The remaining two SNPs *CYP3A4* (rs2740574) and *CYP1A2* (rs2069514) were associated with a decreased risk of developing OUD [7]. *CYP3A4* (rs2242480) and *CYP1A2* (rs3743484) have also been associated with a decreased risk of substance use disorder [10].

As noted above, data related to opioid overdose deaths have been reported relative to race/ethnicity [2, 3]. While race/ethnicity and ancestry have been used interchangeably, race/ethnicity are social constructs, whereas ancestry is biological [11, 12]. The focus of this manuscript is the evaluation of relationships between the identified SNPs and OUD diagnosis in the biogeographical genetic ancestry groups (BGAG) of African American/Afro-Caribbean and European and between males and females [11].

## Material and methods

### Study design

The cross-sectional genetic association study design was previously published [7]. Briefly, consecutive patients were identified via a random selected model across urban-based EDs in three large Ohio cities. Adult individuals over the age of 18 years, who spoke English were eligible for study enrollment. Patients were enrolled over an 18-month period from June of 2020 through November of 2021. The institutional review boards of each participating institution approved the study.

After informed consent was obtained, participants completed a detailed survey assessment to gather self-reported information potentially associated with OUD. This included details of past opioid and other substance use, mental health disorders and other health/medical history. Participants who reported opioid use within the previous year were then directed to respond to questions modeled off the Diagnostic and Statistical Manual of Mental Disorders (DSM-5) criteria for OUD, administered by research staff trained by a licensed psychologist. In participants that reported opioid use beyond at least a year prior, the same modified DSM-5 questions were asked to determine if they may have ever met criteria for OUD. Detailed self-reported race and ethnicity were related to the appropriate BGAG, which are commonly used and reported on in pharmacogenomics [11]. Buccal swabbing was used to harvest cheek cells from each participant.

### Genotyping

The buccal swabs cheek cell samples were shipped to a pharmacogenomics (PGx) testing laboratory (Genemarkers LLC, Kalamazoo, MI) for genotyping related to 180 single nucleotide polymorphisms (SNP) thought to be associated with OUD [13]. Of the 180 variants, 60 were related to pharmacokinetic pharmacogenes (metabolism and transport) and the remainder (120) were related to pharmacodynamic pharmacogenes with a potential association to the dopamine reward pathways [7, 14, 15].

### Methods

**Fig. 1:**
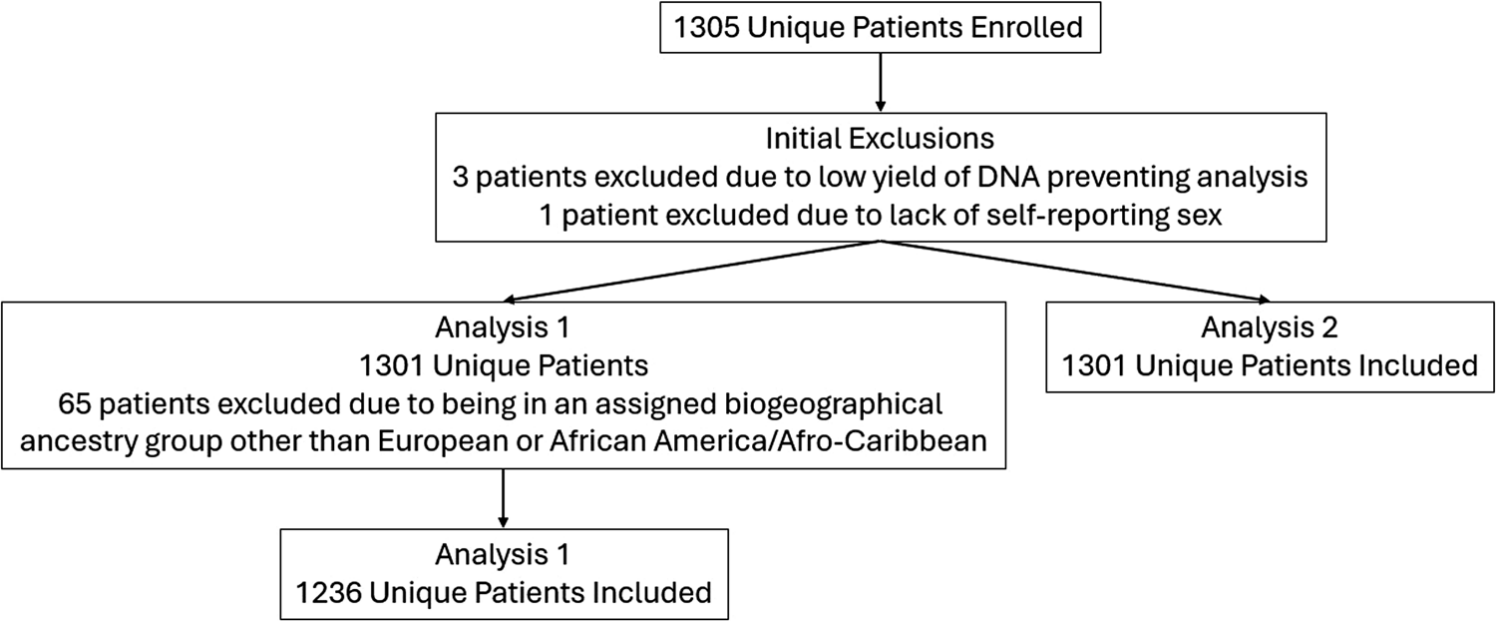
Sub analysis enrollment flow diagram. BGAG is the self-reported assigned biogeographical genetic ancestry group.

Of the 180 variants genotyped in each individual, six variants, rs15524 and rs776746 related to *CYP3A5*, rs2740574 related to *CYP3A4*, rs324029 and rs2654754 related to *DRD3*, and rs2069514 related to *CYP1A2*, which were previously found to be associated with OUD, were investigated for how their association with OUD may be modified [7]. To be included in this analysis, an rs number must have had at least five subjects for each BGAG, variant, and OUD status combinations. Two analyses were completed for demographic information derived from or directly available in the study data. Analysis 1 investigated whether the assigned BGAG derived from self-identified race of participants as African/Afro-Caribbean or European indicated different associations for variant carriers (WT/SNP or SNP/SNP) vs the homozygous wild type (WT/WT) individuals. The related biogeographical genetic ancestry group assignments were based on self-reported race, ethnicity characteristics and sex. There were 1305 patients enrolled in the original study (Fig. 1). Three were excluded due to lack of sufficient sample for DNA analysis and one was excluded for not reporting their sex, leaving 1301 participants. Analysis 1 excluded 65 participants who’s associated BGAG was not African/Afro-Caribbean or European, and one participant who did not self-report their sex (Fig. 1). After exclusion there were 1236 participants available for analysis 1 (Table 1). Analysis 2 investigated whether the self-reported sex at birth (male or female) of participants indicated different associations for the variant carriers (WT/SNP or SNP/SNP) vs the homozygous wild type (WT/WT) individuals. To be included in this analysis, an rs number must have had at least five subjects for sex, variant, and OUD status combinations. For analysis 2, there were 1301 participants. As noted above, after exclusion of one participant who did not provide their self-reported sex there were 1301 participants available for analysis 2 (Table 1). To explore the potential different associations between BGAG (Analysis 1) and sex (Analysis 2) on ever having OUD diagnosed (lifetime; yes/no), we employed multiple logistic regression and adjusted for age in both analyses and sex in analysis 2. Each variant, identified by their respective rs number had a separate multiple logistic regression model for BGAG and sex analyses where each one was an independent variable. A total of 12 multiple logistic regression models were completed. Analyses which had sample sizes of less than five (5) for the BGAG, sex, variant (rs number), and OUD status combination were not considered for interpretation due to small sample size. R statistical software Version 4.0.4 was used to conduct all analysis.

**Table 1:** Characteristics of emergency department patients enrolled in PGx study included in analysis.

		Study patients	Included in analysis
Age (years; mean (SD))		46.1 (15.7)	
Biogeographical Genetic Ancestry Group assigned based on self-reported race (%)	European	637 (49)	1236
	African American/Afro-Caribbean	599 (46)	
	Other	65 (5)	
Sex at birth (%)	Female	697 (53.6)	1301
	Male	604 (46.4)	

### Secondary data power analysis

A secondary data power analysis revealed that a multiple linear regression assessing interactions with a sample size of 1,236 would have at least 80% power to detect an effect size of 0.25. This power analysis corrects for the 180 statistical tests we performed by setting $\alpha =(0.05/180)\,=2.778\,\times 1{0}^{-4}$. Power analyses were completed using G*Power 3.1.9.7.

## Results

### Biogeographical genetic ancestry group, genetics and OUD

**Fig. 2:**
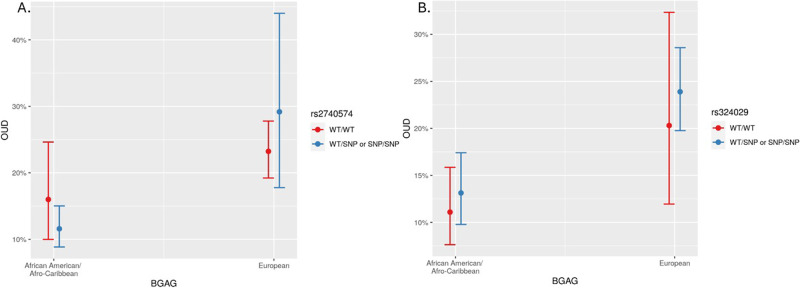
Examples of genetic varaints related to opioid use disorder. Relationship between (**A**) rs2740574 and (**B**) rs324029 and biogeographical genetic ancestry group (BGAG) on opioid use disorder (OUD). Each point on the graph is the mean for the subgroup and the 95% CI (*n* = 1236).

Only two variants, rs2740574 (*CYP3A4*) and rs324029 (*DRD3*), met our criteria for having at least five subjects for all BGAG, variant, and OUD status combinations. Neither variant had statistically significant interactions with either investigated BGAG which were less than the typical *p*-value cutoff of 0.05. Figure 2A illustrates the relationship between rs2740574 and BGAG on OUD while adjusting for sex and age. Variant carriers (WT/SNP or SNP/SNP) in the African/Afro-Caribbean participants had slightly lower predicted probabilities of OUD as compared to wild type (WT/WT) individuals. This relationship was opposite in European ancestry individuals which showed that variant carriers (WT/SNP or SNP/SNP) had slightly higher predicted probabilities of OUD as compared to wild type (WT/WT) individuals. For rs324029, variant carriers (WT/SNP or SNP/SNP) in the African American/Afro-Caribbean and European participants had slightly higher predicted probabilities of OUD as compared to wild type (WT/WT) individuals (Fig. 2B). For reference, the frequencies of the six variants associated with OUD in the study population, including rs2740574 and rs324029, as above, and in those noted in the Genomes Aggregation Database (gnomAD; V3) are presented in Table 2.

**Table 2:** Frequencies of single nucleotide polymorphisms (SNP) found to be related to risk of opioid use disorder in the African American/Afro-Caribbean and European biogeographical genetic ancestry groups (BGAG).

SNP	Position^a^	Reference allele (WT)	Variant allele	Variant frequency in African American/Afro-Caribbean BGAG		Variant frequency in European BGAG	
				Study	gnomAD genome^b^	Study	gnomAD genome^b^
rs15524	99648291	A	G	69.4	58.5	6	7.6
rs776746	99672916	T	C	18	30.5	94.3	93.1
rs2740574	99784473	C	T	23.4	35.9	97.2	96.5
rs324029	1.14E+08	A	G	25.6	34.5	70.2	72
rs2654754	1.14E+08	G	A	63.1	67.5	97.7	97.2
rs2069514	74745879	G	A	31.3	27.7	1.9	1.3

^a^Genome position based on build GRCh38

^b^Based on v3 genome data from the Genomes Aggregation Database (gnomAD) retrieved from the Ensembl API.

### Sex, genetics, and OUD

**Fig. 3:**
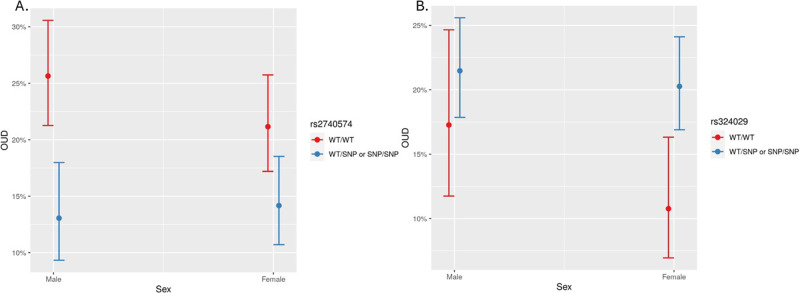
Relationship between (**A**) rs2740574 and (**B**) rs324029 and sex on opioid use disorder (OUD). Each point on the graph is the mean for the subgroup and the 95% CI (*n* = 1301).

All six variants met the criteria for having at least five subjects for all sex, variant, and OUD status combinations. None of the variants had statistically significant interactions with sex which were less than the typical *p*-value cutoff of 0.05. Figure 3 illustrates examples of two variants related to OUD. The relationship between rs2740574 and sex on OUD is shown in panel A while adjusting for sex and age. Variant carriers (WT/SNP or SNP/SNP) in males had decreased predicted probabilities of OUD as compared to wild type (WT/WT) male participants. The observed magnitude of decrease was dampened in female participants which showed that variant carriers (WT/SNP or SNP/SNP) had only slightly lower predicted probabilities of OUD as compared to wild type (WT/WT) participants. In males, having the wild type genotype was statistically associated with 1.78 times the predicted probability of OUD as compared to the males with the SNP variant carriers. For rs324029, variant carriers (WT/SNP or SNP/SNP) in both male and female participants had slightly higher predicted probabilities of OUD as compared to wild type (WT/WT) individuals (Fig. 3 panel B).

## Discussion

The self-reported assigned biogeographical genetic ancestry groups (BGAGs) of African American/Afro-Caribbean and European did not impact the relationship between genetic variants and risk of OUD. Similarly, sex did not influence these relationships. In the current literature, there exists a disparity between BGAGs and the incidence of OUD [14, 15]. This discrepancy has prompted the formulation of a hypothesis that attributes this variance to the presence of specific genetic variations in one BGAG as compared to another [15]. In the present study, we explored this proposition in a predetermined set of SNPs that were previously found to be associated with the risk of OUD [7]. Our findings lacked any statistical evidence supporting an additional distinction between the aforementioned BGAGs with regard to these SNPs. This paucity of evidence may be attributed to the limited sample sizes of the respective groups with different frequencies of variants seen across BGAGs or the absence of any actual distinctions between the BGAGs and the probability of OUD for these specific SNPs [16, 17]. Although this outcome fails to substantiate the underlying hypothesis, it does furnish evidence towards alternate hypotheses involving other SNPs that were not the subject of the present study or the possibility that the difference in question is not further amplified by the BGAG of the individual concerned. During the time of this study, researchers identified a number of SNPs that were not tested for in our study [18–20]. These included *KDM4A* (rs3791033) and *LRRIQ3* (rs640561) in the European BGAG relative to opioid prescription use, *OPRM1* (rs9478500) in the European BGAG, and *OPRM1* (rs1799971 and rs79704991) and *FURIN* (rs11372849) in the European BGAG, relative to opioid addiction, as well as *OPRM1* (rs9478500) in European and African BGAGs, relative to OUD [18–20]. In the present study, the SNPs examined, including the two SNPs where data criteria were met for formal evaluation, rs2740574 (*CYP3A4*) and rs324029 (*DRD3*) are found in different frequencies across BGAGs (Table 2).

The two SNPs identified are seen at considerably lower frequencies in the African American/Afro-Caribbean BGAG (*CYP3A4* rs2740574; 0.359, *DRD3* rs324029; 0.345) as compared to the European BGAG (*CYP3A4* rs2740574; 0.965, *DRD3* rs324029; 0.72). It has been noted that due to lower frequencies of some variants, recruitment of enough patients to have a reasonably powered study can be difficult, if not impossible [21]. Other studies have also found *DRD3* (rs324029) to be associated with an increased risk of OUD in the Han Chinese population [22]. In a cohort of African American patients heterozygous (T/C) or homozygous (C/C) for CYP3A4 (rs2740574) were found to require higher doses of buprenorphine [22, 23].

Recognizing BGAG population differences in the frequency of a given variant can inform what would be expected in an individual from a specific BGAG. This is different from the social construct of race, where an individual’s genetics can be very different from another individual in the same race category. The Human Genome Project noted that, “two random individuals from any one group are almost as different [genetically] as any two random individuals from the entire world” [24, 25]. The use of the BGAGs allows for consistent communication of data from pharmacogenomics studies and presents a clearer way of comparing frequencies of a given variant [11]. However, a limitation of this study was that the related biogeographical genetic ancestry group assignments were based on self-reported race, ethnicity characteristics and sex. In this study, we did not employ analysis of SNPs that were optimal for identifying ancestry as these were not part of the SNP panel related to pharmacokinetics or pharmacodynamics in the context of OUD [26]. It has been reported that there can be significant European contribution in African American populations, and this may have had an impact on our evaluation [27].

Pharmacokinetic and pharmacodynamic differences have been noted between males and females, with an underlying genetic context being autosomal-based with the potential impact of epigenetics [28–31]. Gene-sex relationships have been identified as specific to men or women [29]. Relative to opioid response, four genes, *CYP1A2*, *CYP3A4*, *NAT2*, and *SLC22A2*, were associated in men only [29]. At the variant level, poor pain control in patients receiving codeine or tramadol in men was related to rs1056837 and rs1056836 [29]. The variant rs76026520 was related to poor pain control in both men and women, whereas rs35742686 was related to adverse events across the sexes [29]. Rs35742686 was included in the 180 variant panel in our original study, however, this variant was not identified as having an influence on OUD risk. Differences in DNA methylation in the context of epigenetics was observed in women versus men relative to *SLC6A4*, noting that women could have different expression of the serotonin reuptake transporter, coded for by *SLC6A4* [31]. In the present study, there was no statistically significant (*p* < 0.05) identified relationship between a genetic variant and sex when considering OUD risk. Specifically addressing OUD, a study of over 48,000 Veterans Health Administration patients diagnosed with OUD noted that men and women had different co-morbidities pointing to the need for appropriate treatment of multi-morbidities to decrease relapsing opioid use [32]. Similar findings were observed when considering treatment outcomes citing that treatments addressing co-morbidity behavioral health diseases as well as psychosocial stress may result in more positive outcomes for women with OUD [33]. Certainly, broader variant analysis and further inclusion of women in PGx studies are needed to further elucidate PGx-OUD relationships.

## Conclusions

The biogeographical genetic ancestry group designation of African American/Afro-Caribbean or European, relative to rs2740574 and rs324029 did not influence either variant’s impact on OUD risk. Similarly, sex did not influence the impact of rs15524, rs776746, rs2740574, rs324029, rs2654754, and rs2069514 on OUD risk. These findings suggest that the evaluation of these variants can be utilized to gain insight relative to OUD risk across sexes in African American/Afro-Caribbean or European individuals although further work needs to be done in larger populations of these patients.

## Data Availability

The datasets generated during and/or analyzed during the current study are available from JL (lambejw@ucmail.uc.edu) on reasonable request.
